# Extent of infarct size and microvascular obstruction following unsuccessful reperfusion in patients with ST-segment elevation myocardial infarction

**DOI:** 10.1186/1532-429X-17-S1-P162

**Published:** 2015-02-03

**Authors:** Justyna Rajewska-Tabor, Magorzata Pyda, Aleksander Araszkiewicz, Anna Kociemba, Magdalena Janus, Magdalena Lanocha, Andrzej Siniawski, Stefan Grajek

**Affiliations:** I Cardiology Clinic, University of Medical Sciences in Poznan, Lusowo, Poland; Magnetic Resonance Department, University of Medical Sciences in Poznan, Poznan, Poland

## Background

Impaired microvascular reperfusion (no-reflow) and unsuccessful infarct-related artery (IRA) revascularization are associated with a worse clinical outcome in patients with ST-segment elevation myocardial infarction (STEMI) treated with primary percutaneous coronary intervention (pPCI). Reperfusion can be identified by epicardial or microvascular flow determined by angiography as Thrombolysis in Myocardial Infarction (TIMI) flow grade and a myocardial blush grade (MBG), respectively. Moreover, microvascular obstruction (MVO) determined by CMR is also a well-known predictor of an unfavorable clinical outcome after STEMI.

The aim of the study was to determine the effect of impaired reperfusion estimated by angiography on microvascular obstruction and infarct size (IS) measured by CMR.

## Methods

We examined 85 patients (mean age 59±11 years; 59 males and 26 females) with first STEMI treated with pPCI within 12 hours from symptoms onset. Infarct related artery TIMI flow and MBG were estimated after pPCI. CMR was performed on the 1.5T system within 96 hours after pPCI. Morphology and function of myocardium was estimated by a steady-state free precession (SSFP) sequence. To evaluate the infarct size and MVO, a late gadolinium enhancement (LGE) technique was performed (10-15 min after the administration of Gd-DTPA). Infarct size was defined as an area greater than 50% of the maximal signal intensity within LGE (FWMH - full-width half maximum). MVO was defined as an area of the absence or hypoenhancement of contrast surrounded by LGE. IS and MVO were determined by planimetry and a summation of discs method.

## Results

Revascularization was successful in 41 patients (MBG 3 and TIMI 3) with preserved ejection fraction (EF=56.3±7.8%), IS of 16.92±11.35g and MVO 0.53±1.28g. Patients with suboptimal reperfusion (TIMI<3 and/or MBG<3) had similar EF (52.7±11.7%; ns) but larger IS (31.7±24.83g) and MVO (3.42±6.13g); (p=0.0008 and p=0.004 respectively).

## Conclusions

Infarct size and microvascular obstruction evaluated by CMR in patients with STEMI are associated with impaired reperfusion. Small IS and MVO results are related to a successful reperfusion after pPCI.

## Funding

N/A.

